# Activity-Based Costing of Intensity-Modulated Proton versus Photon Therapy for Oropharyngeal Cancer

**DOI:** 10.14338/IJPT-20-00042.1

**Published:** 2021-06-25

**Authors:** Nikhil G. Thaker, David Boyce-Fappiano, Matthew S. Ning, Dario Pasalic, Alexis Guzman, Grace Smith, Emma B. Holliday, James Incalcaterra, Adam S. Garden, Simona F. Shaitelman, G. Brandon Gunn, C. David Fuller, Pierre Blanchard, Thomas W. Feeley, Robert S. Kaplan, Steven J. Frank

**Affiliations:** 1 Department of Radiation Oncology, The University of Texas MD Anderson Cancer Center, Houston, TX, USA; 2 Arizona Oncology, The US Oncology Network, Tucson, AZ, USA; 3 The Institute for Cancer Care Innovation, The University of Texas MD Anderson Cancer Center, Houston, TX, USA; 4 Harvard Business School, Boston, MA, USA

**Keywords:** proton radiation therapy, IMPT, oropharyngeal carcinoma, IMRT, TDABC, time-driven activity-based costing

## Abstract

**Purpose:**

In value-based health care delivery, radiation oncologists need to compare empiric costs of care delivery with advanced technologies, such as intensity-modulated proton therapy (IMPT) and intensity-modulated radiation therapy (IMRT). We used time-driven activity-based costing (TDABC) to compare the costs of delivering IMPT and IMRT in a case-matched pilot study of patients with newly diagnosed oropharyngeal (OPC) cancer.

**Materials and Methods:**

We used clinicopathologic factors to match 25 patients with OPC who received IMPT in 2011-12 with 25 patients with OPC treated with IMRT in 2000-09. Process maps were created for each multidisciplinary clinical activity (including chemotherapy and ancillary services) from initial consultation through 1 month of follow-up. Resource costs and times were determined for each activity. Each patient-specific activity was linked with a process map and TDABC over the full cycle of care. All calculated costs were normalized to the lowest-cost IMRT patient.

**Results:**

TDABC costs for IMRT were 1.00 to 3.33 times that of the lowest-cost IMRT patient (mean ± SD: 1.65 ± 0.56), while costs for IMPT were 1.88 to 4.32 times that of the lowest-cost IMRT patient (2.58 ± 0.39) (*P* < .05). Although single-fraction costs were 2.79 times higher for IMPT than for IMRT (owing to higher equipment costs), average full cycle cost of IMPT was 1.53 times higher than IMRT, suggesting that the initial cost increase is partly mitigated by reductions in costs for other, non-RT supportive health care services.

**Conclusions:**

In this matched sample, although IMPT was on average more costly than IMRT primarily owing to higher equipment costs, a subset of IMRT patients had similar costs to IMPT patients, owing to greater use of supportive care resources. Multidimensional patient outcomes and TDABC provide vital methodology for defining the value of radiation therapy modalities.

## Introduction

The shift toward value-based cancer care, defined as the quality of health outcomes relative to the cost of achieving those outcomes [1–4], will require understanding the true costs of cancer care delivery. But cost accounting currently used in health care uses charges and reimbursements as proxies for cost [5]. For health care providers, the cost incurred during health care delivery is neither the reimbursement received nor the charge generated; it is the resources actually used to deliver care over the complete care cycle. As reimbursements for health care delivery shift from the existing fee-for-service system, which rewards volume rather than value, to value-based payments, clinicians will need to understand their true costs of delivering care.

Advanced technology in the United States is partially responsible for driving improvements in cancer care outcomes, but those improvements come with potentially steep increases in the cost of cancer care delivery [6–8]. The field of radiation therapy (RT) in particular has relied on costly technologic innovations to improve cancer care outcomes [9]. The evolution from 2-dimensional to 3-dimensional and, more recently, to intensity-modulated radiation therapy (IMRT) has improved outcomes and reduced toxicity for patients so treated [10–12]. The intrinsic physical properties of proton RT allow superior sparing of nontarget tissues distal to the target volume. Intensity-modulated proton therapy (IMPT) holds promise for further sparing healthy tissues from incidental irradiation [13, 14]. Many head and neck tumors, including those of the oropharynx, are found near normal structures such as the brainstem, spinal cord, oral cavity, and salivary glands. Dosimetric and early clinical studies have suggested that the lower doses to these structures, allowed by IMPT, can reduce the incidence and severity of costly and quality-of-life-threatening treatment-related toxicity [15–17]. However, IMPT equipment and operating costs are higher than those for IMRT, a differential that has driven higher reimbursement for proton RT in general and IMPT in particular.

Defining the value of technologic innovations has become critical in the current health care environment. Particularly for costly technologies such as IMPT, implementation of a robust cost-accounting method would significantly strengthen comparative effectiveness analyses, aid in patient-centered decision-making, and provide direct insight into opportunities to maximize value. Several industries have successfully applied the concepts of time-driven activity-based costing (TDABC) [18, 19], a bottom-up cost accounting method that measures the cost of resources used based on the actual time that personnel and equipment are used to treat patients [20–22]. Although TDABC has been applied mostly to industrial personnel and process improvements, this concept can be readily applied to measure and communicate the value of technologically oriented health care delivery systems.

Two recent reports indicate improved dosimetry and reduced need for gastrostomy (feeding) tubes among patients with oropharyngeal cancer (OPC) undergoing IMPT versus IMRT [15, 16, 23]. However, whether these improvements reduce provider costs by reducing resource utilization is unknown. In this analysis, we used this case-control data set to pilot a TDABC analysis to measure the true cost of care delivery over the full cycle of care.

## Materials and Methods

### Patient Selection and Case Matching

Patients with OPC who received definitive IMPT between 2011 and 2012 were identified from a prospectively collected database of patients who received proton therapy for head and neck malignancies at a single, tertiary referral cancer center [15, 16]. Patient data were collected under internal review board–approved protocol. Patients with a history of head and neck radiation, including postoperative RT, were excluded. Information extracted from the records included details on demographics, disease, and treatment and included sex, age, smoking history, primary site (tonsil or base of tongue), presenting T and N status, and receipt of radiation and chemotherapy. Placement of gastrostomy tubes was also recorded. The comparison group was selected by querying an institutional database of 998 patients who received IMRT as definitive therapy for OPC in 2000-09 to identify suitable cases for the matched cohort. Patients from this database were matched in a 1:1 ratio with the 25 patients in the IMPT cohort. Matching was done sequentially based on variables believed to be most relevant to toxicity, including laterality of treatment (ipsilateral versus bilateral), primary site (tonsil versus base of tongue), T category, N category, receipt of concurrent chemotherapy or induction chemotherapy, smoking status, sex, and age. TDABC was used to compare the treatment costs of these 2 case-matched cohorts.

### TDABC Analysis

**Figure 1.**
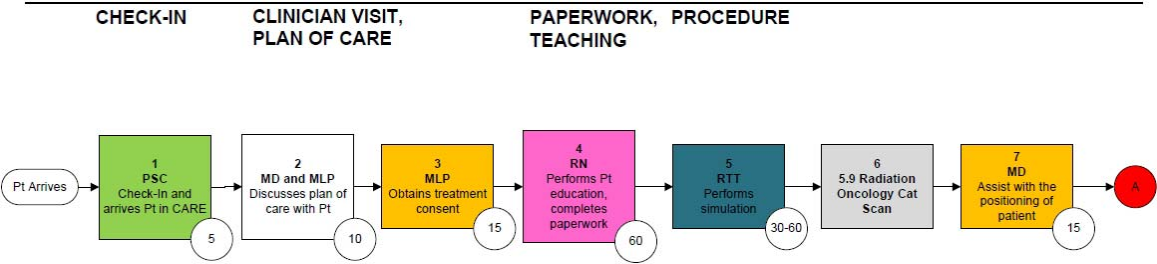
Representative process map of the consultation and simulation workflow. Each step includes a resource (personnel, facility, equipment) and time in minutes. (A) Next steps in the simulation and treatment planning workflows. Numbers in circles indicate numbers of minutes for each step. Abbreviations: Cat, computed tomography; MD, physician; MLP, mid-level provider; PSC, patient services coordinator; Pt, patient; RN, research nurse; RTT, radiation therapist.

Clinical, administrative, and financial teams created patient-level process maps for the full cycle of care, from the initial consultation to 30 days after the completion of RT. Figure 1 represents a portion of the consultation and simulation workflow. These process maps included all ancillary clinical services rendered during the course of RT. Each step in the process map was associated with a specific resource (personnel, facility, or equipment) and the time that resource was used to completely perform each activity [24]. Activity times for each step were documented by content experts, frontline staff, direct observation of personnel, and the institutional scheduling system. Variation in treatment due to patient characteristics were captured with decision and chance nodes within the process maps; the percentage value at each node indicated the probability that a patient would pass through that specific clinical pathway.

TDABC was conducted as described in the literature [24, 25]. Briefly, each activity was associated with a personnel resource. Average hourly rates were calculated for each personnel resource, based on the particular job code. Compensation data based on job codes were obtained from the institutional PeopleSoft (Oracle Inc, Redwood Shores, California) payroll application. Fully allocated costs included direct and indirect costs. Direct costs include the actual resources involved in a patient's care, such as personnel, equipment, facilities, supplies, and support services. Indirect or “overhead” costs associated with patient-facing resources that support these direct services, such as information technology, billing, human resources, and other space or facilities, were allocated as a proportion based on the existing hospital costing approach. These costs were not analyzed via TDABC and were allocated on the basis of existing hospital costing approach. Costs associated with depreciation of RT and diagnostic imaging equipment were also embedded into the cost analysis by using a simple depreciation model based on institutional and manufacturer's recommendations. Practical capacity for equipment was estimated from institutional normal work week and number of hours and procedures per day. The total salary and benefit expense for a particular job group was divided by the annual number of work hours in a year, and adjusted for nonproductive and indirect work time. The adjusted average hourly rate was divided by 60 minutes to calculate the capacity cost rate (CCR). The cost for each step in a process map was therefore calculated by multiplying the time elapsed during the step, in minutes, by the CCR for that resource.

A data relationship management software algorithm was created to link each patient-specific clinical activity (based on CPT codes) with a process map and therefore a TDAB cost. The total TDAB cost for the full cycle of RT (either IMPT or IMRT) was calculated as the sum of all patient activities from initial consultation to 30 days after RT. We further compared all costs to the baseline cost of the least expensive IMRT patient, which we set to 1.0. The TDABC costs were adjusted for inflation to FY2019.

### Statistical Analysis

A χ^2^ test evaluated differences in categorical clinicopathologic variables, the Wilcoxon rank sum test evaluated differences in continuous numeric variables, and the Cochran-Armitage trend test assessed between-group comparisons of ordinal variables. Factorial analysis of variance was used to compare the independent variables of treatment modality and use of gastrostomy tubes to the dependent variable, TDABC. Paired *t* test was used to compare cost distributions between patients treated with IMPT and IMRT. *P* < .05 was considered significant, and all tests were 2-sided.

## Results

As previously reported [15], no significant differences were noted between the 25 patients with OPC treated with IMPT and 25 case-matched patients treated with IMRT (Table), except for age with IMPT patients being significantly older than IMRT patients. Comparison of acute toxicity between groups in the current study revealed that significantly fewer patients in the IMPT group needed gastrostomy tubes (20% versus 48% for the IMRT group, *P* = .035).

**Table.** Patient characteristics for case-matching (in order of priority assigned) between patients with oropharyngeal cancer treated with intensity-modulated proton therapy versus those treated with photon-based intensity-modulated radiation therapy.

Characteristic	No. of patients given IMPT (%)	No. of patients given IMRT (%)	*P* value
Laterality of radiation			1.00
Unilateral	5 (20)	5 (20)	
Bilateral	20 (80)	20 (80)	
Tumor location			1.00
Tonsil	18 (72)	18 (72)	
Base of tongue	7 (28)	7 (28)	
T category			1.00
1	5 (20)	5 (20)	
2	16 (64)	16 (64)	
3	2 (8)	2 (8)	
4	2 (8)	2 (8)	
N category			1.00
0	1 (4)	1 (4)	
1	5 (20)	5 (20)	
2a	2 (8)	2 (8)	
2b	10 (40)	10 (40)	
2c	5 (20)	5 (20)	
3	2 (8)	2 (8)	
Received concurrent chemotherapy	13 (52)	15 (60)	.776
Received neoadjuvant chemotherapy	16 (64)	14 (56)	.773
Smoking status			.848
Current	5 (20)	4 (16)	
Former	7 (28)	6 (24)	
Never	13 (52)	15 (60)	
Sex			.667
Male	23 (92)	21 (86)	
Female	2 (8)	4 (16)	
Age, median (range), y	60 (37-83)	55 (43-74)	.046

**Abbreviations:** IMPT, intensity-modulated proton therapy; IMRT, intensity-modulated radiation therapy.

**Figure 2.**
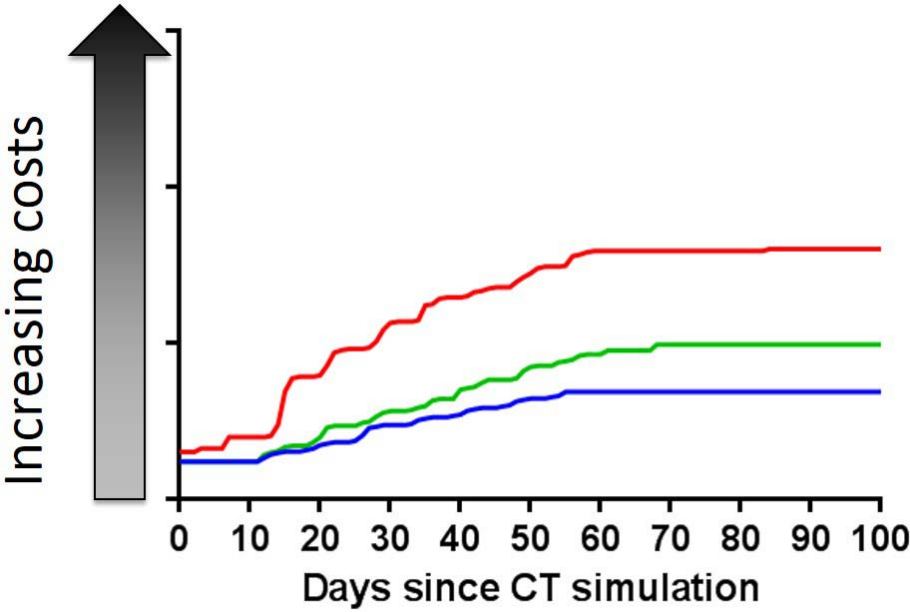
Cumulative time-driven activity-based costs for 3 patients treated with IMRT for oropharyngeal cancer. Although all 3 patients received the same chemoradiation therapy with IMRT, differences in the need for supportive care services, additional imaging due to clinical delays, and other causes led to large variations in cumulative costs over the full care cycle. Abbreviation: IMRT, intensity-modulated radiation therapy.

TDABC analysis was begun for the first 3 patients treated with IMRT as an initial test of the cost calculation algorithm. All 3 patients received the same chemoradiation therapy with IMRT for OPC, but costs varied significantly among them (Figure 2). The 3 largest cost drivers for explaining the large variations in cumulative TDABC over the full cycle of care were supportive care services, emergency room and inpatient admissions, and additional diagnostic imaging due to clinical delays.

**Figure 3.**
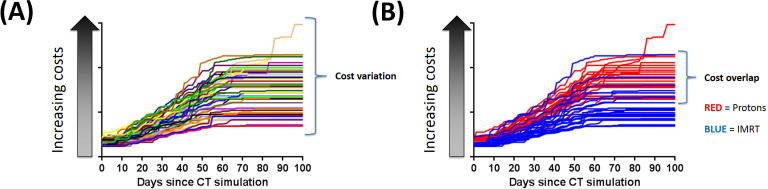
(A) Cumulative time-driven activity-based costs for all 50 patients treated with IMRT or IMPT in this case-matched cohort. (B) Cumulative time-driven activity-based costs for all 50 patients, stratified by treatment modality. Overall, the cumulative costs for 28% of the IMRT patients were higher than for the least-expensive IMPT patient. Abbreviations: IMPT, intensity-modulated proton therapy; IMRT, intensity-modulate radiation therapy.

Costs ranged from 1.0 to 4.3 times the lowest-cost IMRT patient when all 50 patients were compared in aggregate (**Figure 3A**). The TDABC for each daily IMPT treatment was 2.8 times higher than for each daily IMRT treatment, mainly because of the higher equipment costs. However, the average full cycle cost of IMPT was only 1.53 times higher than that for IMRT. Interestingly, 28% of IMRT patient costs overlapped with IMPT patient costs (**Figure 3B**). For patients treated with IMRT, TDABC ranged from 1.00 to 3.33 (mean 1.65, SD 0.56). For patients treated with IMPT, TDABC ranged from 1.88 to 4.32 (mean 2.58, SD 0.39) (*P* < .05). Approximately 47% of all IMRT costs and 65% of all IMPT costs were associated with technology utilization, including simulation and treatment delivery.

**Figure 4.**
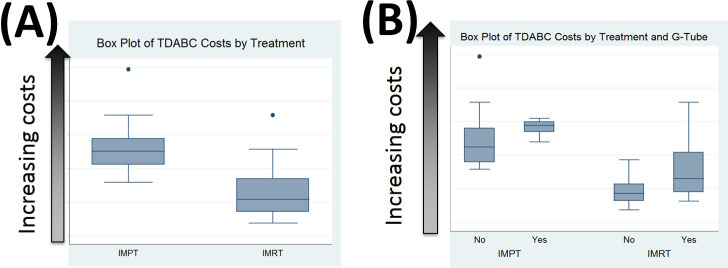
(A) Box plot analysis of time-driven activity-based costs by treatment modality. (B) Box plot analysis of time-driven activity-based costs by treatment modality and the need for gastrostomy (G) tubes.

To further implement the per-patient costing methodology, we then assessed the cost of gastrostomy tubes and the effect of that cost on cumulative TDABC. On average, the need for a gastrostomy tube increased spending by approximately $6000, with overlap in costs for patients treated with IMPT who did not need a tube versus patients treated with IMRT who did need a tube (Figure 4). Cost drivers for patients with gastrostomy tubes included surgery, endoscopy, intravenous hydration, other supportive care services, pharmacy, inpatient stay, and emergency room visits.

## Discussion

As technology continues to advance in service of improving patient care, TDABC can be used to measure true provider costs for cancer care. Although most costing analyses focus on the average or typical costs associated with care delivery, our study includes per-patient analyses, which can provide more subtle and granular costing information regarding the variation in costs inherent with delivering care to individual patients, each of whom has a unique biology and possibly sensitivity to treatments. Although IMPT's higher equipment costs leads to, on average, higher costs than IMRT, we identified a subset of IMRT patients (28%) whose total costs overlapped with IMPT patients. Higher costs arose in part from greater use of supportive resources (such as gastroenterology, emergency and inpatient services). Defining this subset is a crucial step toward understanding not only cost drivers but also clinically challenging disease presentations. Although health care organizations have begun to focus on quality metrics and on outcomes that matter to patients, less emphasis has been given thus far to variations in costs caused by different patient characteristics.

We find that the full cycle costs of IMPT versus IMRT dropped from 2.8x differential per fraction to 1.5x. The difference between these costs includes, in part, the additional costs associated with toxicity. TDABC can therefore be used to uniquely quantify the costs associated with excessive toxicity and can furthermore be incorporated into toxicity burden endpoints. Previous studies have attempted to combine various toxicity endpoints into a single parameter. For instance, Lee et al [26] summarized toxicity data from cancer trials into a toxicity burden score, which included information from weighted sums of multiple lower- and higher-grade toxicities. Similarly, Bayesian designs for clinical trials have also incorporated clinically weighted sums of toxicity scores into a Total Toxicity Burden score [27]. TDABC can be used within this context to measure toxicity-related resource costs (ie, total toxicity costs) at the individual patient level. More direct and effective cost comparisons can therefore be made between the higher upfront costs of innovative technology versus the potential cost reduction associated with lower toxicity. Furthermore, TDABC-measured toxicity costs can be incorporated into Total Toxicity Burden endpoints, including assessments of financial toxicity associated with treatment-related patient expenses (including premiums, copayments, coinsurance, deductibles, lost household income/assets, medical debt, and bankruptcy). Our institution has conducted a randomized trial to assess clinician-reported outcomes, patient-reported outcomes, and TDABC for patients with OPC treated with IMRT versus IMPT, which will continue to define outcome and toxicity differences.

While this study looks at the TDABC costs until 30 days after treatment, one limitation in this study is that the chronic care costs and the subsequent TDABC impact are not included in this analysis. As patients return home from their treatment at our tertiary cancer care center, CCR would have to be defined at each local hospital for the patients in this study. Access to claims data is one approach to further estimate the financial toxicity of side effects and complications from treatment over a longer follow-up period. Such an analysis could help to identify subsets of patients for whom more advanced technology could be a high-value therapy. Without patient-level costing data, we would not have been able to identify the existence of specific cost drivers of such heterogeneity, because the traditional ratios of costs to charges and reimbursements do not accurately portray underlying resource utilization.

We also found that technology (including simulation and treatment delivery) costs accounted for 47% (IMRT) to 65% (IMPT) of the full cycle (including chemotherapy and ancillary services) cost for each patient. Most previous applications of TDABC have pertained to personnel costs [21, 24, 28, 29], which can account for 70% or more of costs in most processes. This discrepancy in radiation oncology emphasizes the need for a cost accounting tool such as TDABC that can accurately aggregate costs along a spectrum of service lines in health care, and appropriately assign reimbursement to procedures that require expensive technology as well as personnel.

This TDABC analysis uncovered several additional value-added opportunities. First, we identified several workflow inefficiencies through process mapping initiatives, including preauthorization, billing, referral, and RT workflows. As providers look to reduce waste in the health care system (eg, nonclinical work that is subsidized by clinical care and inefficient processes), which has been estimated to be as high as 30% to 40% [2], TDABC can help streamline resource utilization by identifying and eliminating non–value-added services and focusing on optimizing care cycle time (rather than optimizing a single step in a long process). This tool thus can be used immediately to identify processes for which initially higher costs could ultimately reduce costs and improve outcomes over the long term. Such an opportunity stems from the improved ability to conduct quality improvement initiatives with TDABC and can complement traditional tools like lean or six sigma initiatives. We also found several opportunities to use this new understanding of the cost drivers of IMPT and IMRT to reorganize several workflows to better optimize resources.

Future analyses will need to account for prospectively matched patients who have been treated with more recent technologies and workflows. Although patients included in this study were treated across a longer period of time, each patient's activities were mapped to the same TDABC process maps and costs, which therefore matches activities to inflation-adjusted costs, including all direct and indirect costs. Additionally, the process for IMPT and IMRT at our institution may differ from other institutions, and TDABC analyses should be re-created specifically for each institution, with institution-specific costs and resources. Finally, owing to the sensitive nature of internal costs, we were unable to publish absolute costs for each of the processes in the workflow, but provide overall relative costs and the absolute costs associated with feeding tube to provide context.

TDABC can also be a useful tool for providers engaging in alternative payment models. TDABC can uniquely enable institution-specific patient-level cost measurement and can provide insight into the actual variation of costs within a cohort of similarly treated patients for treatments with high capital expenditures. Because TDABC analyses are institution-specific, such analyses can provide important internal data regarding cost variations that may not be captured adequately through retrospective review of charges or reimbursements. In turn, these data can contribute to financial analyses that will be needed to ensure that bundled payments are priced adequately and allow for equitable patient access to high-quality, cost-effective care.

## Conclusions

Assessing the value of advanced technologies in cancer treatment is an essential component of our evolving health care delivery system. TDABC is a powerful costing method that can be used to measure the provider's cost of care delivery and provide critical insights into the value of advanced technology. This tool is useful for measuring the comparative costs of competing processes, such as IMPT and IMRT, over entire care cycles and can be implemented at the per-patient level to identify subsets of patients where higher upfront costs may ultimately be of higher value. As payers move toward alternative payment model initiatives, TDABC may also prove to be vital in pricing health care, in engineering high-value alternative payment models, in processing improvement initiatives, and in ensuring equitable and rational patient access to advanced technologies while systematically collecting, measuring, and analyzing standardized outcome and cost metrics.
